# Supplementary material for: FERN – a Java framework for stochastic simulation and evaluation of reaction networks
Source: BMC Bioinformatics. 2008 Aug 29;9:356. doi: 10.1186/1471-2105-9-356 (PMC2553347; doi:10.1186/1471-2105-9-356)
Supplement: Additional file 1 — FERN distribution, Version 1.3. This archive contains the FERN source code and binaries as well as documentation and example models in FernML and SBML. [file 1471-2105-9-356-S1.zip › fern/doc/javadoc/fern/simulation/observer/class-use/GnuPlotObserver.html]

Uses of Interface fern.simulation.observer.GnuPlotObserver


---


|  |  |  |
| --- | --- | --- |
| **Overview** \| **Package** \| **Class** \| **Use** \| **Tree** \| **Deprecated** \| **Index** \| **Help** | |  |
| PREV   NEXT | **FRAMES**    **NO FRAMES**     **All Classes** |  |


---


## **Uses of Interface fern.simulation.observer.GnuPlotObserver**

| Packages that use GnuPlotObserver | |
| --- | --- |
| **fern.simulation.observer** | Provides classes to observe certain aspects of simulations. |

Uses of GnuPlotObserver in fern.simulation.observer

| Classes in fern.simulation.observer that implement GnuPlotObserver | |
| --- | --- |
| `class` | `AmountAtMomentObserver`             Observes the amount of some molecule species at a certain moment. |
| `class` | `AmountIntervalObserver`             Observes amounts of molecule species repeatedly after certain intervals. |
| `class` | `FireTypeObserver`             Observes the different types of firings. |
| `class` | `IntervalObserver`             Base class for observing certain aspects repeatedly after a given interval. |
| `class` | `LeapObserver`             Observes the amount of given molecule species at each `step`. |
| `class` | `MultiAmountIntervalObserver`             Observes amounts of molecule species repeatedly after certain intervals. |
| `class` | `RandomNumberGeneratorCallObserver`             Observes the calls to the random number generator and count the number of drawn random number of different distributions. |
| `class` | `ReactionIntervalObserver`             Observes number of firings of reactions repeatedly after certain intervals. |

---


|  |  |  |
| --- | --- | --- |
| **Overview** \| **Package** \| **Class** \| **Use** \| **Tree** \| **Deprecated** \| **Index** \| **Help** | |  |
| PREV   NEXT | **FRAMES**    **NO FRAMES**     **All Classes** |  |


---
